# What is the best fixation method in medial patellofemoral ligament reconstruction? A biomechanical comparison of common methods for femoral graft attachment

**DOI:** 10.1051/sicotj/2024004

**Published:** 2024-02-08

**Authors:** Léonard Vezole, Stanislas Gunst, Laure-Lise Gras, Jobe Shatrov, Ozgur Mertbakan, Sébastien Lustig, Elvire Servien

**Affiliations:** 1 Orthopaedics Surgery and Sports Medicine Department, FIFA Medical Center of Excellence, Croix-Rousse Hospital, Lyon University Hospital 69004 Lyon France; 2 Univ Lyon, Claude Bernard Lyon 1 University, IFSTTAR, LBMC UMR_T9406 F69622 Lyon France; 3 LIBM – EA 7424, Interuniversity Laboratory of Biology of Mobility, Claude Bernard Lyon 1 University Lyon France

**Keywords:** Biomechanical evaluation, Cadaveric study, Medial patellofemoral ligament, Patellar instability

## Abstract

*Introduction*: A variety of techniques have been described for femoral fixation in medial patellofemoral ligament reconstruction (MPFLr). The aim of this study was to compare the biomechanical performance of the most used methods for graft fixation in the femur using human cadaveric tissue. We wondered what is the best fixation method for femoral fixation in MPFL reconstruction? *Hypothesis*: A suspensory fixation device provides the best femoral fixation. *Material and method*: Twenty cadaveric knees were tested. Four femoral fixation methods were compared (5 knees per group): interference fixation with a Biosure© RG 5 mm and a 7 mm, suture anchor (Healicoil Regenesorb 4.75 mm ©) and suspensory fixation with the Ultrabutton©. The testing was divided in preconditioning, cyclic loading and load to failure. Load to failure, elongation, stiffness and mode of failure were recorded and compared. *Results*: The Ultrabutton© had the highest mean ultimate load (427 ± 215 N (*p* = 0.5)), followed by Healicoil anchor © (308 ± 44 N (*p* > 0.05)) and the interference screw of 7 mm (255 ± 170 N (*p* > 0.05)). Mean stiffness was similar in the Ultrabutton© and 4.75 mm. Healicoil anchor © groups (111 ± 21 N/mm and 119 ± 20 N/mm respectively), and lowest in 7 mm Biosure© screw fixation group (90 ± 5 N/mm). The Biosure© 5 mm RG screw presented 100% of premature rupture because of tendon slippage. The Ultrabutton© presented the lowest premature rupture (40%). *Discussion*: A suspensory fixation for the femur had the lowest number of graft failures and highest load to failure. This study has implications for surgeons’ choice of graft fixation in MPFLr. It is the first study to test the most commonly femoral used fixation methods, allowing direct comparisons between each method.

## Introduction

Medial patellofemoral ligament reconstruction (MPFLr) is the most performed surgery for lateral patellar instability [1–3]. Commonly a two-bundle technique is utilized with either a semitendinosus or gracilis autograft to fashion two free limbs that replicate the broad attachment site of the native MPFL (broad insertion) on the patella [4–9].

Multiple methods exist for bony attachment of the graft to the patellar and femoral sites in MPFLr as screw fixation, endobutton, suture anchors [5, 6, 10–14] and soft tissue fixation [15]. Several studies have examined the biomechanical performance of fixation devices for the patella [5, 10, 11, 16–19], less for the femur [20, 21] and in some studies, it has been simultaneously tested (femur and patella) limiting interpretation of individual fixation methods [22–25]. The biomechanical strength of femoral fixation in MPFLr has been studied less. This is despite the fact it is perhaps the most important of the two to consider as it is the more likely site to fail in MPFLr [26].

This study aimed to compare the biomechanical strength of commonly described graft-fixation methods for femoral graft anchor sites in MPFLr performed using a double bundle hamstring technique in human cadaveric tissue. The hypothesis was that the suspensory fixation provides the best femoral fixation.

## Materials and methods

### Patients

This cadaver study was approved by the IRB (Institutional Review Board) of the University. A total of 20 fresh-frozen cadaveric knees were obtained from an anatomical program at our institution (10 matched pairs; 7 females, 3 males; mean age 87.5 ± 12.5). None of the cadavers studied had a history of bone or soft tissue injury, surgery, or osteoporosis. Specimens were randomly assigned to one of four groups for femoral fixation. Allocation was performed so that when one knee was designated to a group, the opposite knee from the same specimen was assigned to another group.

### Specimen preparation

An anteromedial approach was used to harvest semitendinosus autografts which was used for femoral testing. This has previously been described for MPFLr with satisfactory results [12, 27]. Once the tendons had been fixed and the bone fully prepared, specimens were stored at −20 °C and thawed at room temperature for 24 h prior to testing.

### Femoral fixation

**Figure 1:**
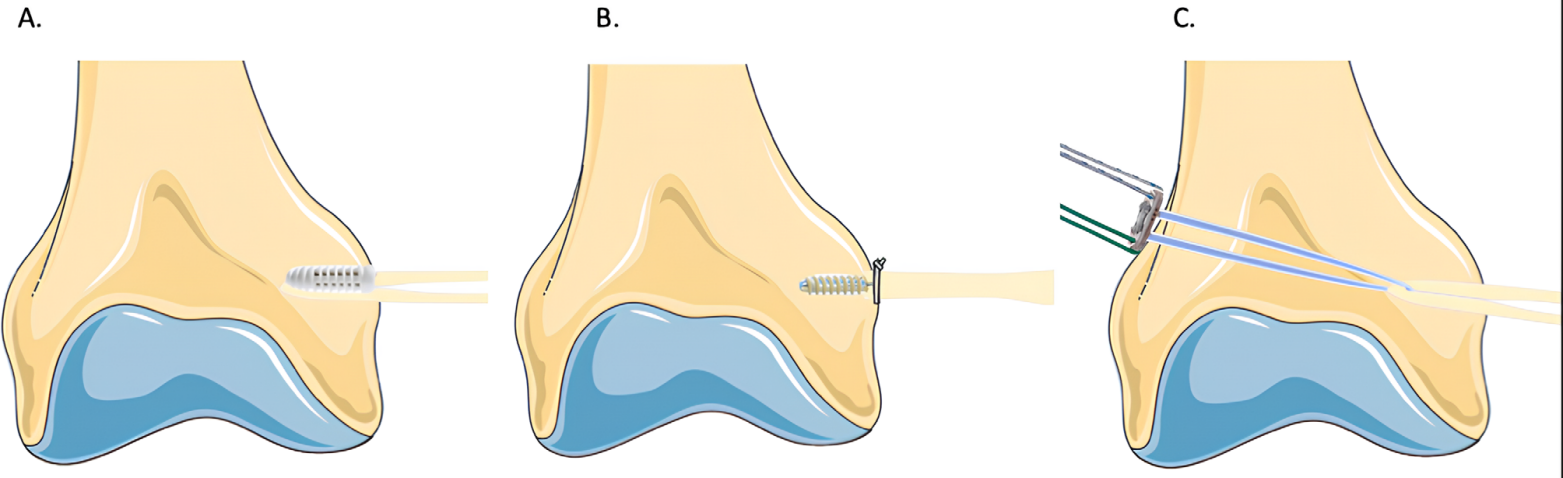
Femoral fixation techniques. (A) Groups F1, F2, interference fixation with a 5 mm for F1 and a 7 mm screw for F2 (Biosure© RG 5 mm and Biosure© RG 7 mm). (B) Group F3, anchor fixation (Healicoil Regenesorb 4.75 mm Anchor). (C) Group F4, suspensory fixation using an endobutton (Ultrabutton©).

The femur was transected 30 cm from the joint line and dissected free of all soft tissue attachments except the insertion of the native MPFL. Femoral fixation methods are summarized in Figure 1.

In group F1 a 5 mm interference screw was used (S5-F) (Biosure© RG 5 mm). A K wire was placed in the middle of the native MPFL insertion and following the creation of a 4.5 mm bicortical tunnel, a 5 mm cannulated drill was used to create a socket 30 mm deep. The end of the graft which was marked at 30 mm was passed through the tunnel and fixed with a 5 × 20 mm interference screw.

In group F2, a 7 mm interference screw was used (S7-F) (Biosure© RG 7 mm). The same steps were followed but the socket was drilled to 30 mm depth using a 7 mm diameter cannulated drill and the graft was fixed with a 7 × 20 mm interference screw.

In group F3, an anchor was used (Healicoil Regenesorb 4.75 mm Anchor ©). A pilot hole was created. and the anchor placed in the center of the anatomical footprint of the MPFL. Next, the graft was secured using 6 alternating half-hitch knots of No. 2 Ultrabraid (Smith & Nephew).

In group F4 an endobutton was used (Ultrabutton©). A K-wire was placed in the center of the anatomical footprint of the MPFL and a 4.5 mm cannulated drill was used to create a bicortical femoral tunnel. The end of the graft was sized and marked at 30 mm. A femoral socket was drilled to a 30 mm depth and to a diameter that was matched to the size of the graft. The graft, with the endobutton loaded, was passed through the femoral tunnel while counter tension was applied. The button was flipped and verified by direct inspection to ensure it was seated flush with the lateral cortex of the femur, and the ends tied once the graft had been shuttled to a depth of 30 mm (as marked on the tendon).

## Methods

### Biomechanical testing

Specimens’ bony parts were embedded into a custom-made metallic pot containing a fixative solution (Polyuréthane-84). Free ends of grafts were then linked to the testing machine with a specific clamp. A 55 mm length of graft was kept, which corresponds to the anatomic length of the MPFL [28]. Tests were executed based on a worst-case scenario with the tension line parallel to the anchors, screws, and the tunnel.

### Methods of assessment

#### Testing protocol

The grafts were tested using an Instron machine – INSTRON 8802 (High Wycombe, England). All tests were filmed at 50 Hz with a PHOTRON SA3 black and white camera (Tokyo, Japan). The testing protocol was divided into three steps: preconditioning, cyclic loading, and load to failure. During the preconditioning phase, 10 loading-unloading cycles between 0 and 20 N were applied to the specimens at 1 Hz. During cyclic loading, 1000 cycles between 20 N and 100 N were applied at 1 Hz. Finally, loading of the specimens up to failure was performed at the constant velocity of 6 mm/min. During the whole test, load (N) and displacement (mm) were recorded with a 1000 N load cell (accuracy of 0.5%) and an LVDT sensor (accuracy of 1%) respectively. Elongation (mm) was measured after cycling. Stiffness (N/mm) was calculated as the slope of the linear portion of the load-displacement curve. Load to failure (N) was extracted from the experimental data. The failure mode was directly observed and recorded on video. Failure was defined as rupture of either the graft or pullout of the fixation device and was considered early if it occurred during cycling, and late failure if it occurred during load-to-failure testing.

#### Statistical analysis

Statistical analysis was performed using SPSS version 25.0 (IBM). Mean and standard deviation values were reported for descriptive statistics. Mean ultimate loads and stiffness were compared between groups using the Kruskall–Wallis and independent Samples t-tests method. Significance was set at <0.05.

## Results

Twenty femoral specimens underwent biomechanical testing. Four methods of fixation were tested creating 4 groups of 5 knees. These results are summarized in Table 1.

**Table 1:** Biomechanical comparison of Femoral fixation methods.

Femoral fixation	Premature rupture during cycling	Ultimate load (N) (Mean ± SD)	Elongation (mm) (Mean ± SD)	Stiffness (N/mm) (Mean ± SD)
Group F1: Biosure© RG 5 mm 5 mm	5/5 (100%)			
Group F2: Biosure© RG 7 mm	2/5 (40%)	255 ± 170	6.7 ± 4.5	90 ± 5
Group F3: Healicoil Regenesorb 4.75 mm Anchor ©	3/5 (60%)	308 ± 44	7.4 ± 1.4	119 ± 20
Group F4: Ultrabutton©	2/5 (40%)	427 ± 215	10.7 ± 3.8	111 ± 21
*P*-value		0.506	0.389	0.208

### Femoral fixation

Over the 20 femoral tests, 12 graft failures occurred early during cycling and were excluded from the statistical analysis.

### Failure mode

**Figure 2:**
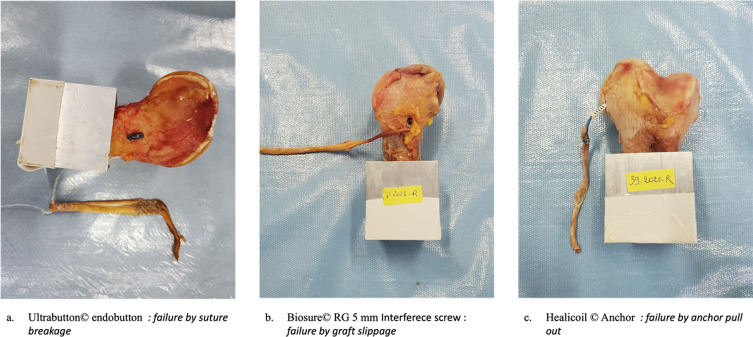
Record of graft failure modes. (A) Suspensory fixation device Ultrabutton© failure by suture breakage. (B) Interference screw Biosure© RG failure by graft slippage. (C) Suture anchor Healicoil Regenesorb 4.75 mm failure by anchor pull-out.

The failure mode of the reconstruction is represented in Figure 2. Seven early failures occurred because of tendon slippage, (all five Biosure© RG 5 mm and two with Biosure RG 7 mm ©), three because of anchor failure (Healicoil Regenesorb 4.75 mm Anchor ©), and two with Ultrabutton© (one passed through the lateral cortex and one had a suture breakage). During load-to-failure testing, three specimens failed by graft rupture (one Biosure© RG 7 mm and two with Ultrabutton©), two by anchor failure (two Healicoil Anchor ©), two by tendon slippage (Biosure© RG 7 mm) and one Ultrabutton© had a suture breakage.

### Graft elongation

**Figure 3:**
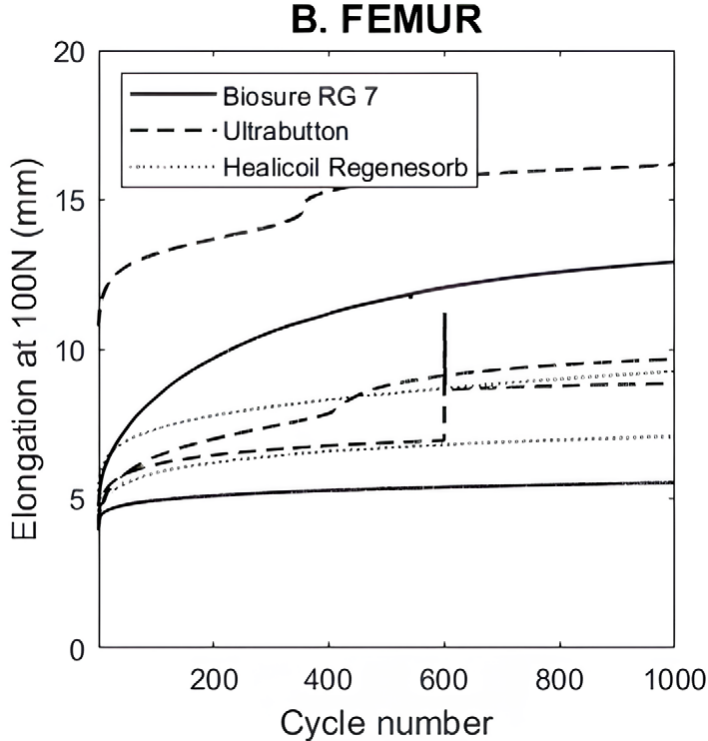
Biomechanical testing results for graft elongation of femoral fixation methods.

Graft elongation after cycling is represented in Figure 3. It was greatest in the endobutton group (10.7 ± 4 mm) and lowest in the interference fixation group with the 7 mm Biosure© screw (6.7 ± 5 mm) and in the Healicoil anchor © group (7.4 ± 1 mm).

### Stiffness

**Figure 4:**
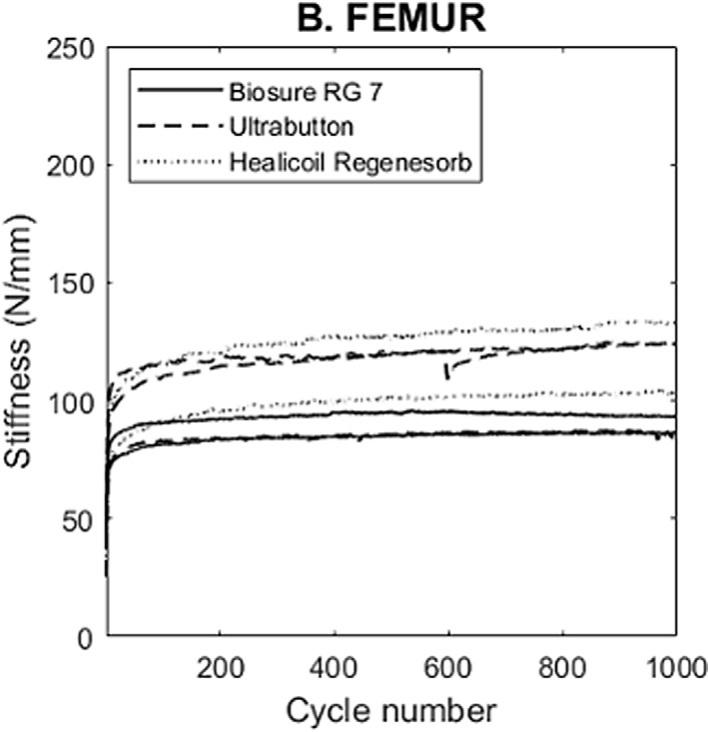
Biomechanical testing results for stiffness of femoral fixation methods.

The evolution of the stiffness during cycling is represented in Figure 4. Regarding Stiffness at 1000 cycles, mean stiffness was similar in the endobutton and 4.75 mm Healicoil anchor groups (111 ± 21 N/mm and 119 ± 20 N/mm respectively), and lowest in the 7 mm Biosure© screw fixation group (90 ± 5 N/mm).

### Load to failure

**Figure 5:**
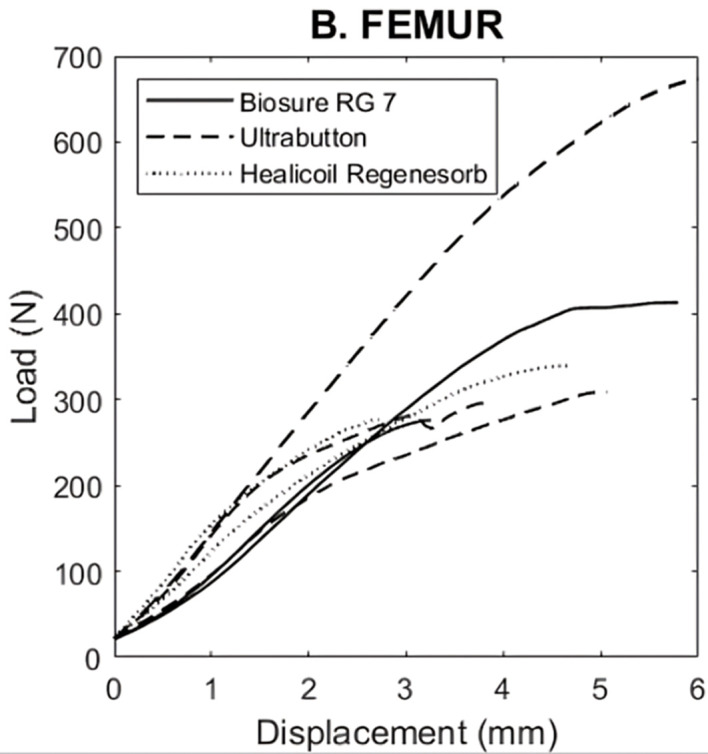
Biomechanical testing results for ultimate load of femoral fixation methods.

The comparison of ultimate load to failure between groups is summarized in Figure 5. The endobutton (Ultrabutton©) had the highest mean ultimate load (427 ± 215 N; *p *> 0.05) and interference fixation with the 7 mm Biosure© RG screw, the lowest (255 ± 170 N; *p* > 0.05). Healicoil anchor © presented a mean ultimate load of 308 ± 44 N (*p* > 0.05).

## Discussion

The most important finding of this study was that a suspensory fixation device for the femur (endobutton) provided the most robust strength of MPFL graft fixation as demonstrated by the lowest number of early failures and highest load to failure. The interference screw of 7 mm for the femoral fixation provided graft behavior that most closely resembled previously described values of the native MPFL. This is not the first study to examine these fixation devices, however, it is the first study to test the most commonly femoral-used fixation methods, allowing direct comparisons between each method in a human cadaver. Indeed, previous literature has usually compared two techniques [20, 21, 29]. Only one study compared anchors, interference screw, and suspensory cortical fixation but this study was performed using an allograft tendon with a single bundle reconstruction and tested femoral and patella attachments simultaneously rather than isolating individual sites [22].

The findings of the present study demonstrated that most of the common fixation methods are stronger than the tensile strength of the native MPFL which has been shown to range from 178 ± 46 N to 208 ± 90 N [16, 23]. The endobutton fixation provided more than double the strength of the native MPFL (427 ± 215 N), followed by the Healicoil suture anchors and the 7 mm Biosure screw.

### Fixation failure modes

The most common mode of failure with anchors was pull out, whereas slippage was the commonest mode of failure for interference screw fixation. Despite the same fixation protocol being used for 7 mm and the 5 mm interference screws, the rate of premature rupture was 100% in the interference screws of 5 mm. The increased diameter of the interference screw has not been correlated to the reduction of graft slippage previously [30, 31].

### Graft elongation

The present study demonstrated no statistically significant difference between fixation methods regarding graft elongation. For femoral fixation, the suspensory fixation showed the greatest elongation (10.7 mm) and the 7 mm interference screw the shortest elongation (6.7 mm). It is unknown if the small differences of 3–4 mm between the methods tested have clinically meaningful effects on outcomes. Joyner et al. showed that approximately 40 mm translation is required for patellar subluxation [22]. Given the unclear effects of elongation and the small differences between methods in this study, it appears the methods studied provide similar results for graft elongation for MPFLr.

### Stiffness

Stiffness (change in length/ultimate load) of the native MPFL has previously been reported to vary from 8 to 12 N/mm [26, 32]. In the current study, all fixation methods demonstrated higher stiffness than the native MPFL and there was no significant difference between techniques. For the femoral reconstruction, interference fixation with a 7 mm screw gave the closest stiffness value (90 N/mm) to the native MPFL. Previous studies have described a wide range of stiffnesses in graft construct, which may be due to differences in cycling protocol, study methodology, and definitions. A construct that has less stiffness may be less susceptible to over-tensioning or be more affected by small changes in tunnel position [32, 33].

### Femoral fixation load to failure

In the current study, the Ultrabutton had the highest ultimate load to failure (427 ± 215 N) and the least number of premature failures. Gould et al reported results comparing femoral interference screw to anchor fixation and found a mean load to failure of 294.0 N for interference screw and 250.0 N for anchor fixation [9]. In the present study, the anchor group had a mean load to failure of 308 ± 44 N and interference screw fixation of 255 ± 170 N. Joyner et al. similarly showed a combination of patellar interference screw fixation and femur suspensory cortical fixation to the highest mean load to failure of all combinations tested [22].

### Limitations

This study has several weaknesses. The number of failures during cyclic loading reduced the sample size in the statistical analysis. However, the number of early failures was consistent with previous studies that have reported 60% early failure and is reflective of the nature of this testing and still provide important information about the utility of these fixation methods [6]. This study analyzed time zero strength, and any subsequent healing could not be considered. The tendons were not first subjected to a pre-loading before being fixed which prevented us from evaluating the mechanical properties of the hamstring alone. But this is by our clinical practice as the grafts are not pre-loaded during MPFLr surgery, before fixation. Furthermore, an inherent problem with all cadaveric testing is that the behavior of tissue may differ *in vivo,* and in 3 femoral MPFLr failure was due to the tendon rupture and not the failure of the fixation method. This may be related to the average age of specimens which was older than that of patients who would normally undergo MPFLr and to the freezing thawing process that may alter the tendon biomechanical properties. However, it is difficult to obtain younger cadaveric tissue and the specimens used in this study are comparable to the literature. There were more females than males in the cadaveric testing, and while the specimens did not have a history of osteoporosis, bone mineral density in post-menopausal females is known to be less than for aged-matched males which has affected the test results. A final limitation is that the linear pull-out test used to evaluate reconstructions in the current study does not consider the normal movement and stresses of the patellofemoral joint. Linear testing, however, represents a worst-case scenario for the forces that would be applied to the graft *in vivo*.

### Significance

This study has clinical relevance to surgeons when considering the choice of graft fixation in MPFLr. No statistically significant difference was demonstrated in the biomechanical performance of the evaluated femoral fixation methods in terms of maximum load to failure, stiffness, and elongation.

## Conclusion and clinical application

This study has implications for surgeons’ choice of graft fixation in MPFLr. It is the first study to test the most commonly femoral-used fixation methods, allowing direct comparisons between each method in a human cadaver. If the surgeon wants to limit the risk of graft failure, he should choose a suspensory fixation for the femur (endobutton), which gives the lowest number of graft failures and the highest load to failure. If the surgeon wants to reach the native behavior of the MPFL he should use a 7 mm interference screw which ensures the closest rigidity to the MPFL.

## Data Availability

Data are available on request from the authors.

## References

[1] Otsuki S, Okamoto Y, Murakami T, et al. (2018) Patellofemoral reconstruction for patellar instability with patella alta in middle-aged patients: Clinical outcomes. Orthop Traumatol Surg Res 104, 217–221. 29410197 10.1016/j.otsr.2018.01.003

[2] Chouteau J (2016) Surgical reconstruction of the medial patellofemoral ligament. Orthopaedics & Traumatology: Surg Res 102, S189–S194. 10.1016/j.otsr.2015.06.030 26797001

[3] Arendt EA (2009) MPFL reconstruction for PF instability. The soft (tissue) approach. Orthopaedics & Traumatology: Surg Res 95, 97–100. 10.1016/j.otsr.2009.09.002 19896428

[4] Deie M, Ochi M, Sumen Y, et al. (2005) A long-term follow-up study after medial patellofemoral ligament reconstruction using the transferred semitendinosus tendon for patellar dislocation. Knee Surg Sports Traumatol Arthrosc 13, 522–528. 15968532 10.1007/s00167-005-0641-x

[5] Hapa O, Aksahin E, Ozden R, et al. (2012) Aperture fixation instead of transverse tunnels at the patella for medial patellofemoral ligament reconstruction. Knee Surg Sports Traumatol Arthrosc 20, 322–326. 21678092 10.1007/s00167-011-1582-1

[6] Hinterwimmer S, Imhoff AB, Minzlaff P, et al. (2013) Anatomical two-bundle medial patellofemoral ligament reconstruction with hardware-free patellar graft fixation: technical note and preliminary results. Knee Surg Sports Traumatol Arthrosc 21, 2147–2154. 23575650 10.1007/s00167-013-2498-8

[7] McNeilan RJ, Everhart JS, Mescher PK, et al. (2018) Graft choice in isolated medial patellofemoral ligament reconstruction: a systematic review with meta-analysis of rates of recurrent instability and patient-reported outcomes for autograft, allograft, and synthetic options. Arthroscopy 34, 1340–1354. 29366741 10.1016/j.arthro.2017.11.027

[8] Panni AS, Alam M, Cerciello S, et al. (2011) Medial patellofemoral ligament reconstruction with a divergent patellar transverse 2-tunnel technique. Am J Sports Med 39, 2647–2655. 21868688 10.1177/0363546511420079

[9] Neri T, Philippot R, Carnesecchi O, et al. (2015) Medial patellofemoral ligament reconstruction: clinical and radiographic results in a series of 90 cases. Orthop Traumatol Surg Res 101, 65–69. 25530480 10.1016/j.otsr.2014.09.023

[10] Raoulis VA, Zibis A, Chiotelli MD, et al. (2021) Biomechanical evaluation of three patellar fixation techniques for MPFL reconstruction: Load to failure did not differ but interference screw stabilization was stiffer than suture anchor and suture-knot fixation. Knee Surg Sports Traumatol Arthrosc 29(11), 3697–3705. 33386885 10.1007/s00167-020-06389-4

[11] Russo F, Doan J, Chase DC, et al. (2016) Medial patellofemoral ligament reconstruction: fixation technique biomechanics. J Knee Surg 29, 303–309. 26190788 10.1055/s-0035-1554922

[12] Schottle P, Schmeling A, Romero J, Weiler A (2009) Anatomical reconstruction of the medial patellofemoral ligament using a free gracilis autograft. Arch Orthop Trauma Surg 129, 305–309. 18704468 10.1007/s00402-008-0712-9

[13] Servien E, Fritsch B, Lustig S, et al. (2011) In vivo positioning analysis of medial patellofemoral ligament reconstruction. Am J Sports Med 39, 134–139. 20929935 10.1177/0363546510381362

[14] Shatrov J, Colas A, Fournier G, et al. (2022) Tibial tuberosity osteotomy and medial patellofemoral ligament reconstruction for patella dislocation following total knee arthroplasty: A double fixation technique. SICOT-J 8, 23. 35699459 10.1051/sicotj/2022023 PMC9196027

[15] Bremond N, Prima R, Rabattu P-Y, et al. (2022) Isolated MPFL reconstruction with soft tissue femoral fixation technique in 54 skeletally immature patients: Clinical outcomes at 2 years follow-up. A French multicenter retrospective study. Orthop Traumatol Surg Res 109(8), 103530. 36565744 10.1016/j.otsr.2022.103530

[16] He W, Yang YM, Liu M, et al. (2013) Reconstruction of the medial patellofemoral ligament using hamstring tendon graft with different methods: a biomechanical study. Chin Med Sci J 28, 201–205. 24382220 10.1016/s1001-9294(14)60002-x

[17] Lenschow S, Schliemann B, Gestring J, et al. (2013) Medial patellofemoral ligament reconstruction: fixation strength of 5 different techniques for graft fixation at the patella. Arthroscopy 29, 766–773. 23395115 10.1016/j.arthro.2012.12.004

[18] Russ SD, Tompkins M, Nuckley D, Macalena J (2015) Biomechanical comparison of patellar fixation techniques in medial patellofemoral ligament reconstruction. Am J Sports Med 43, 195–199. 25261087 10.1177/0363546514550992

[19] Zhao X, Zhang H (2021) Biomechanical comparison of 2 patellar fixation techniques in medial patellofemoral ligament reconstruction: transosseous sutures vs suture anchors. Orthop J Sports Med 9(10), 23259671211041404. 34692878 10.1177/23259671211041404 PMC8529315

[20] Gould HP, Delaney NR, Parks BG, et al. (2021) Interference screw versus suture anchors for femoral fixation in medial patellofemoral ligament reconstruction: a biomechanical study. Orthop J Sports Med 9(3), 2325967121989282. 33763498 10.1177/2325967121989282 PMC7944534

[21] Johnston TR, Liles J, Riboh J (2020) Anchor-based femoral fixation for physeal-sparing medial patellofemoral ligament reconstruction: a time-zero biomechanical comparison with tenodesis screw fixation. Am J Sports Med 48, 3021–3027. 32909820 10.1177/0363546520951523

[22] Joyner PW, Bruce J, Roth TS, et al. (2017) Biomechanical tensile strength analysis for medial patellofemoral ligament reconstruction. Knee 24, 965–976. 28684171 10.1016/j.knee.2017.04.013

[23] Mountney J, Senavongse W, Amis AA, Thomas NP (2005) Tensile strength of the medial patellofemoral ligament before and after repair or reconstruction. J Bone Joint Surg Br 87, 36–40. 15686235

[24] Zampieri A, Girardin C, Hocquet B, et al. (2022) Patellar dislocation recurrence after pediatric MPFL reconstruction: Bone tunnels and soft tissues versus suture anchors and interference screw. Orthop Traumatol Surg Res 109(8), 103515. 36528262 10.1016/j.otsr.2022.103515

[25] Tsushima T, Tsukada H, Sasaki S, et al. (2019) Biomechanical analysis of medial patellofemoral ligament reconstruction: FiberTape^®^ with knotless anchors versus a semitendinosus tendon autograft with soft anchors. J Orthop Sci 24, 663–667. 30573394 10.1016/j.jos.2018.11.018

[26] Amis AA, Firer P, Mountney J, et al. (2003) Anatomy and biomechanics of the medial patellofemoral ligament. Knee 10, 215–220. 12893142 10.1016/s0968-0160(03)00006-1

[27] Ridley TJ, Macalena JA, Arendt EA (2018) Isolated medial patellofemoral ligament reconstruction with semitendinosus tendon allograft. JBJS Essent Surg Tech 8, e5. 30233977 10.2106/JBJS.ST.17.00033 PMC6143300

[28] Luxenburg D, Rizzo MG (2023) Anatomy, bony pelvis and lower limb: medial patellofemoral ligament. Treasure Island (FL), StatPearls Publishing, 35593802

[29] Tsushima T, Tsukada H, Sasaki S, et al. (2019) Biomechanical analysis of medial patellofemoral ligament reconstruction: FiberTape(R) with knotless anchors versus a semitendinosus tendon autograft with soft anchors. J Orthop Sci 24, 663–667. 30573394 10.1016/j.jos.2018.11.018

[30] Micucci CJ, Frank DA, Kompel J, et al. (2010) The effect of interference screw diameter on fixation of soft-tissue grafts in anterior cruciate ligament reconstruction. Arthroscopy 26, 1105–1110. 20678709 10.1016/j.arthro.2009.12.022

[31] Namkoong S, Heywood CS, Bravman JT, et al. (2006) The effect of interference screw diameter on soft tissue graft fixation. Bull Hosp Jt Dis 63, 153–155. 16878837

[32] Elias JJ, Cosgarea AJ (2006) Technical errors during medial patellofemoral ligament reconstruction could overload medial patellofemoral cartilage: a computational analysis. Am J Sports Med 34, 1478–1485. 16685097 10.1177/0363546506287486

[33] Bollier M, Fulkerson J, Cosgarea A, Tanaka M (2011) Technical failure of medial patellofemoral ligament reconstruction. Arthroscopy 27, 1153–1159. 21664791 10.1016/j.arthro.2011.02.014

