# Erratum to: “What is the best fixation method in medial patellofemoral ligament reconstruction? A biomechanical comparison of common methods for femoral graft attachment”

**DOI:** 10.1051/sicotj/2024015

**Published:** 2024-05-16

**Authors:** Léonard Vezole, Stanislas Gunst, Laure-Lise Gras, Jobe Shatrov, Özgür Mert Bakan, Sébastien Lustig, Elvire Servien

**Affiliations:** 1 Orthopaedics Surgery and Sports Medicine Department, FIFA Medical Center of Excellence, Croix-Rousse Hospital, Lyon University Hospital 69004 Lyon France; 2 Univ Lyon, Claude Bernard Lyon 1 University, IFSTTAR, LBMC UMR_T9406 69622 Lyon France; 3 LIBM– EA 7424, Interuniversity Laboratory of Biology of Mobility, Claude Bernard Lyon 1 University 69622 Lyon France

Regarding this article, published on February 8, 2024, an error occurred in one of the author’s name:

The correct name of the fifth author is Özgür Mert Bakan

The Publisher will ensure that all modifications will be correctly transmitted to the indexation bases.

The Publisher apologizes for this mistake.

